# Live Hot, Die Young: Transmission Distortion in Recombination Hotspots

**DOI:** 10.1371/journal.pgen.0030035

**Published:** 2007-03-09

**Authors:** Graham Coop, Simon R Myers

**Affiliations:** 1 Department of Human Genetics, University of Chicago, Chicago, Illinois, United States of America; 2 Broad Institute of MIT and Harvard, Medical and Population Genetics, Cambridge, Massachusetts, United States of America; Fred Hutchinson Cancer Research Center, United States of America

## Abstract

There is strong evidence that hotspots of meiotic recombination in humans are transient features of the genome. For example, hotspot locations are not shared between human and chimpanzee. Biased gene conversion in favor of alleles that locally disrupt hotspots is a possible explanation of the short lifespan of hotspots. We investigate the implications of such a bias on human hotspots and their evolution. Our results demonstrate that gene conversion bias is a sufficiently strong force to produce the observed lack of sharing of intense hotspots between species, although sharing may be much more common for weaker hotspots. We investigate models of how hotspots arise, and find that only models in which hotspot alleles do not initially experience drive are consistent with observations of rather hot hotspots in the human genome. Mutations acting against drive cannot successfully introduce such hotspots into the population, even if there is direct selection for higher recombination rates, such as to ensure correct segregation during meiosis. We explore the impact of hotspot alleles on patterns of haplotype variation, and show that such alleles mask their presence in population genetic data, making them difficult to detect.

## Author Summary

Recombination is a fundamental component of mammalian meiosis, required to help ensure that daughter cells receive the correct complement of chromosomes. This is highly important, as incorrect segregation causes miscarriage and disorders such as Down syndrome. In addition to its mechanistic function, recombination is also crucial in generating the genetic diversity on which natural selection acts. In humans and many other species, recombination events cluster into narrow hotspots within the genome. Given the vital role recombination plays in meiosis, we might expect that the positions of these hotspots would be tightly conserved over evolutionary time. However, there is now considerable evidence to the contrary; hotspots are not frozen in place, but instead evolve rapidly. For example, humans and chimpanzees do not share hotspot locations, despite their genomic sequences being almost 99% identical. The explanation for this may be, remarkably, that hotspots are the architects of their own destruction. The biological mechanism of recombination dooms them to rapid extinction by favoring the spread of hotspot-disrupting mutations. By mathematically modeling human hotspot evolution, we find that this mechanism can account for fast hotspot turnover, and in fact makes it very difficult for active hotspots to arise at all. Given that active hotspots do exist in our genome, newly arising hotspots must somehow be able to bypass their self-destructive tendency. Despite their importance, it is difficult to identify mutations that disrupt hotspots, as they hide their tracks in genetic data.

## Introduction

There is now compelling evidence, from sperm studies and from the analysis of patterns of genetic variation, that the general pattern of recombination in humans is highly nonuniform throughout the genome [1–4]. Both of these approaches have shown that a large proportion of crossing over is restricted to small regions, so-called recombination hotspots (typically 1–2kb wide), where crossing over occurs much more frequently than in the surrounding region. This heterogeneity of rate is clearly an important factor in determining the association between alleles along the genome, and therefore an understanding of the forces controlling hotspot occurrence and evolution would greatly benefit many analyses that employ variation data.

A number of studies have found that fine-scale patterns of recombination are poorly conserved between humans and our nearest relative, the chimpanzee [5–8]. That is, hotspots are present in both species, but largely in different genomic locations. Consistent with the idea of rapid evolution of hotspots through time, historical estimates of the rate of recombination at a number of hotspots were found to be inconsistent with their present day intensity in sperm by Jeffreys et al. (2005) [9]. Many hotspots must be transient features of the genome, which suggests that hotspots currently present within the population might frequently be polymorphic, a possibility not incorporated in most current models of evolution.

A possible explanation of hotspot transience is the phenomenon of biased gene conversion. In essence, the idea is that an allele that locally disrupts a hotspot may have an unequal probability of transmission in individuals heterozygous for the disrupting allele. Both current models and empirical observations of recombination (both discussed further below) strongly suggest that typically we expect this transmission bias to favor transmission of the hotspot-*disrupting* allele [10]. The result is an increase in the probability of this allele being driven to fixation in the population, resulting in the elimination, or strong reduction in intensity, of the hotspot. Boulton et al. (1997) [11] observed that biased transmission should lead to the elimination of hotspots from the genome over time. The reasons for the survival of hotspots in the face of this drive are unclear, and Boulton et al. (1997) [11] described this problem as the “recombination hotspot paradox.” Boulton et al. (1997) [11] and Pineda-Krch and Redfield (2005) [12] investigated possible resolutions of the hotspot paradox via simulation. Both studies found that the proposed benefits of recombination, e.g., breakup of deleterious combinations of mutations or ensuring correct segregation, are insufficient to maintain a hotspot in the presence of driven disrupting alleles.

There is already direct evidence that biased gene conversion in favor of hotspot-disrupting alleles occurs at several specific human hotspots. A number of authors have investigated particular hotspots in male meiosis using sperm studies (see Carrington and Cullen (2004) [13] for a review). Jeffreys and Neumann (2005) [14] and Jeffreys and Neumann (2002) [15] showed that in two well-characterized human hotspots, DNA2 and NID1, respectively, variation at particular SNPs appeared to affect hotspot activity. In each case, one of the two alleles strongly suppressed hotspot activity, and was overtransmitted in heterozygotes. An earlier study also found a signal in the data that strongly suggested a similar phenomenon operating at the human MS32 hotspot [16]. Given the necessarily low number of individuals analyzed in sperm studies at any one hotspot, and the very small number of human hotspots that have been investigated in this manner, it seems likely that such “hotspot alleles” segregating in the population at large are common. This phenomenon has also been observed at a hotspot in mice [17] and has been studied with artificial alleles in yeast (e.g., see [10,18,19]).

The study of the phenomenon of hotspot evolution in humans is of particular interest. Many features of recombination are highly conserved across eukaryotes and so it is likely that much of the work of Boulton et al. (1997) [11] and Pineda-Krch and Redfield (2005) [12] on the hotspot paradox will hold true in many organisms. There are, however, some known features that may be more specific to our species. First, the fact that hotspots are visible from patterns of linkage disequilibrium (LD) implies that they must exist in particular locations for tens of thousands of generations, contrasting with the lack of sharing between humans and chimpanzees [5–8]. Second, humans have a relatively small effective population size. This means that genetic drift will play a key role in the fate of alleles that alter our local recombination landscape.

Motivated by the above observations, our aim here is to study the effect of transmission bias at hotspot-influencing mutations on human hotspot evolution. Throughout, we consider realistic human parameters, and the human-specific features described above. In particular, we address, in separate sections, three specific and important questions regarding the properties of recombination hotspots.

First, how long do we expect a typical hotspot to persist in the population? This quantity is key to understanding the proportion of hotspots that should be shared between humans and chimpanzees. It also enables us to ask whether all, or nearly all, hotspots should be shared between human populations. Second, for plausible human population dynamics, which models of how hotspots arise are compatible with the observed spectrum of human hotspot intensities? Third, what is the effect of alleles that disrupt or enhance a hotspot on diversity patterns within a population? A signal for disrupting or enhancing alleles would allow these alleles to be identified, helping to reveal more about the mechanisms of double-strand break (DSB) initiation.

Boulton et al. (1997) [11] and Pineda-Krch and Redfield (2005) [12] use fully simulation-based approaches to consider the effect of biased transmission on the fate of hotspots under a range of models and parameters. Our work builds on this, but differs in several key respects. First, we develop an analytical framework that fully allows for the effect of drift and biased transmission on hotspots. This permits intuition regarding the effect of changes in model parameters, and allows rapid calculation of results. Second, there has been a rapid accumulation of data on the human recombination landscape. This enables us to focus strongly on realistic parameter values. We present a dynamic picture of evolving hotspots in humans and suggest solutions to the hotspot paradox in humans.

In this article, we consider a general setting in which primary sequence changes can disrupt, or introduce, hotspots. As described above, such hotspot-influencing mutations have been found at an appreciable fraction of studied human hotspots [14–16]. The factors that control the location and heat of hotspots remain far from completely understood. Work in yeast suggests that both local nucleotide sequence and larger features of chromosome structure are involved [20], and we are beginning to learn more about the control of human hotspots. Recent work [4] has found strong evidence that particular sequence motifs are overrepresented in hotspot locations. Further, the two hotspot SNPs whose alleles suppress hotspot activity [14,15] disrupt two of these motifs, providing compelling evidence that the motifs directly influence these hotspots in *cis* [4]. Although the mechanism of hotspot disruption or introduction is not the focus of our study, the mutation (or creation) of recombination-promoting motifs demonstrates one way in which this can occur.

## Results

### Biological Processes of Recombination and Gene Conversion

We consider biased gene conversion in terms of the DSB repair model [21]. This is the working model of recombination in yeast [20], and is likely to apply similarly to mammals [1]. We stress that our results are not dependent on this particular model, only on the empirical observation of biased transmission in hotspots in the species of interest. However, the DSB model does offer a natural biological explanation for biased gene conversion at hotspots, and so, for the sake of completeness, we offer a brief description here of this model and its implications.

Under the DSB model, recombination occurs during meiosis as a result of a DSB at a site. The break occurs on either the maternal or paternal chromosomes. During the repair process, information at sites immediately flanking the break site is lost. The other chromosome remains intact, and must supply the missing information via gene conversion to repair the break, so that whenever a DSB occurs, the offspring carries the genetic material of the unbroken copy in a region immediately surrounding the break.

DSBs are processed by specific repair machinery to produce one of two outcomes. The first possibility is gene conversion. Here, one of the parental chromosomes is present for all but a short tract flanking the DSB site, where the information is copied from the intact chromosome. The second possibility, gene conversion accompanied by crossing over, results in the chromosomal material in the offspring on one side of the crossover being derived from the maternal parental chromosome and the material on the other side being from the paternal parental chromosome. Note that even when crossing over occurs, it is accompanied by gene conversion repair of the DSB.

**Figure 1:**
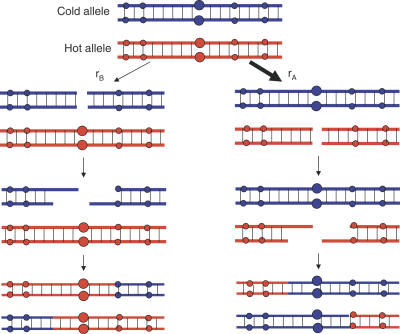
A Simple Representation of the Occurrence and Resolution of a DSB into a Crossover Event during Meiosis In a heterozygote for a hotspot and nonhotspot allele (large red and large blue dots respectively), two nonsister chromatids of the four chromatids present at meiosis are shown. A DSB on the chromatid bearing (red) or not bearing (blue) the hotspot allele occurs with probabilities *r*_A_ and *r*_B_, respectively. In this case, the material immediately surrounding the DSB is lost, and in repairing the break by gene conversion (accompanied by crossing over in this example) the sequence from the other, nonsister, chromatid is used. This results in a bias towards transmission of the non-hotspot allele, proportional to the difference *r*_A_ − *r*_B_ in initiation rates. The figure could also be drawn to show gene conversion without crossing over, which does not result in exchange of markers (smaller dots) flanking the DSB.

Imagine two alleles, A and B, at a particular locus where allele B reduces the rate of DSBs in *cis,* so that the haplotypes containing the A allele are more often subject to DSBs. In an AB heterozygote, gene-conversion repair of DSBs will cause transmission to be biased in favor of allele B. Therefore, any segregating site or allele able to prevent the local occurrence of DSBs on the chromosome is automatically favored by biased gene conversion (see Figure 1).

### Modeling the Frequency of a Hotspot Allele

We start by constructing a model to describe the frequency through time (the frequency trajectory) of a segregating allele that influences the heat of a hotspot. In this model, the frequency of the allele will change through time due to random genetic drift, as well as both biased gene conversion and mutation. The population genetic behavior of models of biased gene conversion has previously been studied by a number of authors [22,23]. We present an analogous model changing the parameters to describe a recombination hotspot.

At a locus L, two allelic classes, A and B, are present. During meiosis in an AB heterozygote, a chromosome in the A class initiates a DSB with probability *r*_A_ and a chromosome in the B class initiates a DSB with probability *r*_B_. Note that the frequency trajectory of the alleles A and B does not depend upon the DSB rate in the homozygotes, as biased transmission does not occur in these individuals. Thus, our model can apply to the case in which rates in heterozygotes are a nonadditive function of the homozygote rates. Without loss of generality we assume that *r*_A_ > *r*_B_, and accordingly sometimes refer to A as the “hotspot” allele and B as the “disrupting” allele. The difference in the rate of initiation of DSBs between the two alleles is denoted by *r*_H_, i.e., *r*_H_ = *r*_A_ − *r*_B_. Using just a two-allele system simplifies reality, since there may in practice be multiple alleles at the locus corresponding to differing DSB initiation rates, but this assumption helps us build a simple model that nevertheless provides insight into the evolution of hotspots.

It is important to note that the rate of DSB formation at the hotspot is distinct from the crossover rate, since many DSBs may result in gene conversion that is not accompanied by crossover. Thus, our parameters reflect the rate of DSB initiation at the hotspot, which can be several times greater than the rate of crossing over in some human hotspots [24]. When a DSB is initiated then with probability *p,* the allele that initiated the DSB is transmitted. Any value of *p* between 0 and 1/2 is biologically possible, with *p* = 0 corresponding to initiation always occurring very close to L, and *p* = 1/2 to no bias.

Potentially, a mutation at any of a number of sites could disrupt the hotspot, and we assume that the chance of a particular site mutating again to allow the hotspot to recover is negligible. We assume also that any such mutation results in a change of rate to *r*_B_, so that mutant chromosomes always become members of the B class. This leads to a simple model of one-way mutation in which only mutation out of the hotspot allele (i.e., from the A class to the B class) is possible. When the A allele is transmitted to the offspring chromosome, with probability μ_D_, the allele mutates to the non-hotspot allelic class B. We are deliberately vague here about the exact relationship of the sites that control the hotspot to the hotspot itself. This stems from our wish to retain generality, particularly given the current incomplete knowledge about the exact mechanism of hotspot disruption.

To simplify the analysis, we assume a constant-size random-mating population with discrete generations (i.e., the standard Wright-Fisher model) and without selection, although these assumptions could be relaxed. The census size of the population is *N,* and the effective size of the population is *N*_e_. The effective population size quantifies the magnitude of genetic drift in a population; the larger the effective population size, the smaller (or slower) the effect of genetic drift. We make use of various methods employed to study similar population genetics models; more specifically, we use the diffusion limit of the Wright-Fisher Model, in which time is rescaled in units of 2*N*_e_.

For large *N*_e_, a model describing the frequency of an allele experiencing biased gene conversion is equivalent to a model of selection with no dominance [23] (genic selection; see Text S1 section 1 for details). If the hotspot allele is undertransmitted (*p* < 1/2), then the hotspot allele effectively acts as a deleterious mutation (although it is not maladaptive). Thus, it is clear that just as the properties of selected alleles in a population are in part governed by the effective size of that population, the effective population size will also affect the properties of alleles that locally influence the heat of recombination hotspots.

In this model, the distortion away from non-Mendelian segregation in heterozygotes is 2*r*_H_(1/2 − *p*) in favor of the hotspot-disrupting allele. We refer to 2*r*_H_(1/2 − *p*) as the drive coefficient. The drive coefficient is equivalent to the selection coefficient in a model of genic selection. As in many population genetics models, we are interested in the relative strength of the drive compared to genetic drift. This is quantified by the product of the effective population size and the drive parameter:

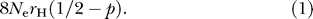
This population scaled drive parameter is equivalent to the population scaled selection parameter 4*N*_e_ *s* in a model of genic selection. Where we do not explicitly consider variation in *r*_H_, the drive parameter 2*r*_H_(1/2 − *p*) will hereafter be denoted by *g,* and the population scaled drive parameter by 4*N*_e_ *g*.

### Human Parameters

Having developed a model of biased transmission in hotspots, we can estimate relevant parameters for humans. Although relatively little is known about the general fine-scale properties of hotspots in humans, a number of sperm-based studies have investigated crossover and gene conversion rates in particular hotspots. The rate of *crossover* in (male) human hotspots so far characterized by sperm studies varies by over two orders of magnitude, ranging on autosomes from the DNA1 hotspot, which has crossing over activity of 0.5 × 10^−5^ Morgans (crossover events per male meiosis), to the DNA3 hotspot, which has 130 × 10^−5^ Morgans [24], and as high as 370 × 10^−5^ for the SHOX pseudoautosomal hotspot [24]. The strength of drive for or against an allele is actually determined by the rate of gene conversion repair of DSBs rather than simply the rate of crossover at the site of the allele. This rate is much more difficult to measure than the crossover rate, since detecting highly localized gene conversion products is more difficult when crossover does not occur. However, conversion without crossing over was estimated by Jeffreys and May (2004) [24] to be four to 15 times more likely to result from a DSB than gene conversion accompanied by crossing over, based on examining three known human hotspots. The level of unaccompanied gene conversion might vary between hotspots, and the frequency of conversion at particular markers declines rapidly with distance from the hotspot center [24]. Typically, however, we expect the drive due to conversion unaccompanied by crossover to be as strong as, or even stronger than, drive due to crossover.

In Table 1 we present plausible estimates of human drive parameters (*g*), based on these studies, for various known human hotspots, including the hottest and coldest identified so far. See Text S1 section 2 for full details of how these estimates are obtained. Although there is considerable difficulty in estimating the drive parameter for particular hotspots and hotspot-disrupting alleles, this analysis suggests that a wide parameter range (0–200) for the drive parameter is likely to encompass the majority of human hotspots.

**Table 1:**
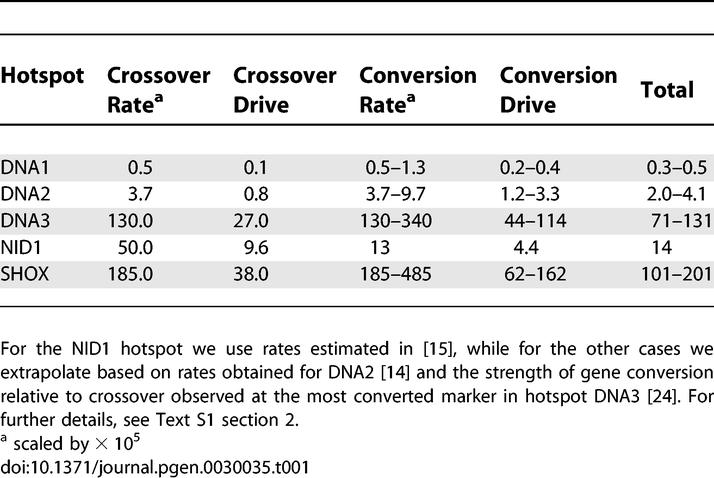
Estimated Magnitude of Drive Parameters Corresponding to an Example Set of Human Hotspots, Based on N_e_ = 10,000

The rate of mutations that disrupt hotspots (μ_D_) is unknown. The per base mutation rate in humans is on the order of 10^−8^ per generation [25]. Clearly, it is probable that only certain mutations within the hotspot will strongly affect its heat. This implies for 1–2kb hotspots that μ_D_ is no larger than 2 × 10^−5^ per generation, resulting in a population scaled mutation rate 4*N*_e_μ_D_, of less than 1 (and probably much less). Throughout the paper, we will make use of results that assume such a relatively low population scaled mutation rate. While this slightly reduces generality, we feel that it allows a clear insight into the role of drive in the evolution of hotspots, and it seems likely that biologically plausible parameters will fall within this range.

### How Long Does a Hotspot Survive?

Suppose two species diverged *T* generations in the past. What fraction of ancestral hotspots ought we to see conserved in both species to the present? A hotspot is most likely to be conserved in both species in the present day if it was fixed in the ancestral species/population, and so we concentrate on the survival time of a hotspot initially fixed in a population. The probability of a hotspot surviving unaffected in a particular species to the present day is the probability of no hotspot-disrupting allele reaching fixation in the population. We will assume that the mutation rate towards the disrupting allele is low enough that only one mutation that disrupts the hotspot is present at an appreciable frequency within the population at any one time. Further, we assume that both the census and effective population sizes are constant since the two populations split.

Alleles that disrupt a hotspot are introduced into the population at rate 2*N*μ_D_. Using a standard population genetic approximation (see Text S1 section 3), we can approximate the probability of no disruptive alleles having arisen and fixed in either population by

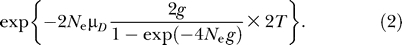
A helpful way to look at the effect of drive on the survival of probability of hotspots is to suppose a mutation at any one of *k* sites could disrupt the hotspot (i.e., let μ_D_ = *k*μ_s_, where μ_s_ is the per site mutation rate). We need not consider exactly how this disruption takes place; it need not occur by a change within any particular sequence motif. We only require that there is some collection of sites capable of removing the hotspot.

Humans and chimpanzees differ on average at about 1.23% of homologous sites [26]. This represents a 0.0123 probability that a neutral allele has fixed since the time of divergence of human and chimpanzee. This means that at a single site, more than 100 mutations (for *N* > 10,000) will have occurred in one species or the other during the time since divergence, but the vast majority will be lost by genetic drift. Within a hotspot, the bias transmission acts upon this introduced variation, and in the case of an intense hotspot, this dramatically increases the probability that one of these 100 mutations will reach fixation.

**Figure 2:**
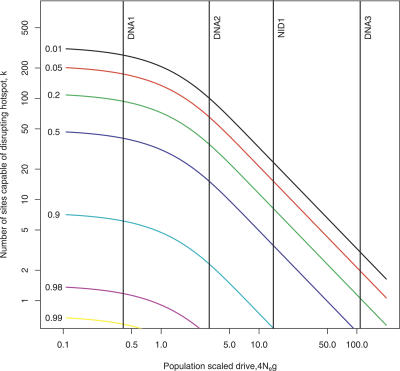
Probability That a Hotspot is Shared between Human and Chimpanzee Contour lines show the probability of sharing a hotspot between human and chimpanzee, as a function of the scaled drive 8*N*_e_ *r*_H_(1/2 − *p*) against the hotspot, and the number of sites capable of disrupting the hotspot, assuming effective population size *N*_e_ = 10,000. Each line refers to a different probability of sharing. The vertical lines correspond to our estimates of likely drive parameters for four known human hotspots, DNA1–3 and NID1. See text for further details.

Given the 1.23% level of neutral divergence, we can calibrate μ_s_ *T* accordingly. Assuming a constant effective population size of 10,000 in both species, we can plot the number of disrupting sites needed to give a fraction α of sharing between the species, for different values of α and different hotspot intensities (Figure 2). As can be seen in Figure 2, the biased gene conversion would strongly influence the chance of survival of DNA3, NID1, and DNA2, and offers an explanation for the observed lack of sharing of hotspots of such heat between the species [5–8]. For a hotspot as intense as DNA3, only 2–3 sites where hotspot-disrupting mutations can arise are needed to reduce the chance of this surviving to the present day in both species to 10%. However, the drive has little effect on relatively weak hotspots such as DNA1. This hotspot would need over 180 sites where disrupting mutations could arise to lower the survival probability to the same level. We therefore expect many more weak hotspots to be shared between humans and chimpanzees compared with more intense hotspots.

This provides a testable hypothesis, although this prediction is currently difficult to test (weak hotspots are harder to identify using population genetic data). The validity of this hypothesis depends on the historical value of *N*_e_ between humans and chimpanzees. A (much) higher ancestral population size, as has been proposed by other authors [27–29], could result in far less sharing for *all* hotspots.

### Is Drive Compatible with the Observed Spectrum of Human Hotspot Intensities?

The “recombination hotspot paradox” [11], asks how any hotspots can arise or persist in the population, if biased gene conversion acts against them. One possible resolution is that if enough mutations introducing hotspots occur that at any given time, a reasonable number of hotspots will be present at high frequency in the population due to simple stochastic drift, despite drive against them. In this section, we explore whether this mutational input offers an explanation for observed human hotspot distributions. First, we examine how many hotspots we expect there to be in a region, if any newly arisen hotspot allele is at a disadvantage due to biased gene conversion in favor of the non-hotspot allele. Later, we consider possible alternatives to this scenario.

In order to gain insight into the number of hotspots present in the population, and their distribution of intensities, we make several simplifying assumptions. We assume that alleles that introduce a hotspot experience the same biased transmission against them as alleles switching off such a hotspot experiences in its favor. This assumption is relaxed later. We also assume that hotspots evolve independently of one another; and that mutation towards hotspots is sufficiently rare that any two hotspot-causing mutations create distinct hotspots.

Hotspot alleles that experience a biased transmission *g* against them are introduced by mutation into the population at rate *N*_e_μ_H_ per generation. Then the expected number of such hotspot alleles with a population frequency in the small interval [*x, x* + *dx*] is

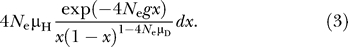
This formula is equivalent to the often-used frequency spectrum of a selected allele [30] (see Text S1 section 4). The number of hotspot alleles within the population depends on the relative rates of introduction of hotspots of different heats, μ_H_, which is difficult to estimate sensibly. For simplicity, we assume that hotspot alleles of any heat are introduced at the same rate, i.e., μ_H_ is not a function of *g*. If recombination were mutagenic (e.g., Hellmann et al. (2003) [31]), then the mutation rate μ_D_ away from hotspots would be expected to be increasing with *r*_H_ and hence with *g,* implying that intense hotspot alleles are even less likely to achieve high frequency in the population, or to be shared between human and chimpanzee.

**Figure 3:**
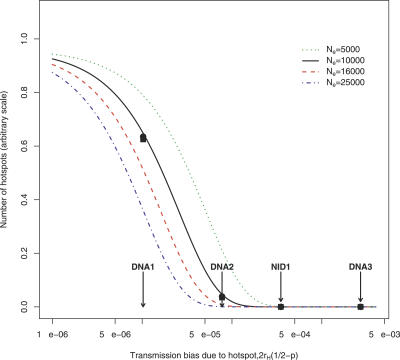
Effects of Demography and Population Size on the Distribution of Hotspot Heats The figure shows the relative expected number of hotspots in the population with frequency above 0.5 for a number of different population sizes and two different demographic models (for low rates of mutation towards [μ_H_ = 10^−8^] and away [μ_D_ = 2.5 × 10^−7^] from the hotspot allele). Each line refers to a different effective population size in the range 5,000–25,000. The black squares show results from Wright-Fisher simulations of a European population size model (*N*_e_ = 10,659) with a bottleneck beginning 1,600 generations ago and reducing the population size 2.5-fold for 1,600 generations, and the black circles show simulation results under a bottleneck model with a 10-fold reduction for 400 generations (for more details of these simulations, see Text S1 section 5). The arrows mark our estimated values for the unscaled drive coefficients at hotspots of the intensity of DNA1–3 and NID1.

Most hotspot alleles will be lost quickly from the population due to genetic drift and drive; only hotspot alleles that reach appreciable frequency in the population will leave a signal in LD data. To examine the number of hotspots that affect LD patterns in a detectable way, we arbitrarily define “visible” hotspots to have a frequency above a set frequency (*y*). The total expected number of such hotspots with frequency greater than *y* is then

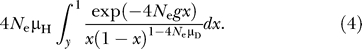
Plotted in Figure 3 is the expected number of hotspot alleles, with frequency >0.5, for a range of effective population sizes and drive coefficients, 2*r*_H_(1/2−*p*). The range of effective population sizes used in Figure 3 were chosen to encompass those likely for humans and chimpanzees. We also performed simulations for plausible human population bottleneck scenarios (see Figure 3 and Text S1 section 5 for full details), as well as for a more accurate approximation of the relationship between hotspot heat and that observed in LD data (Text S1 section 6) and find that our results remain essentially unchanged.

The first point to note is that considering mutations that create hotspots, and drift, does go some way towards solving the “hotspot paradox” of Boulton et al. (1997) [11], since we do see some level of hotspots present in the population at high frequency. However, the number of hotspots at a given frequency drops off exponentially with the drive against them (i.e., their heat), and in larger populations intense hotspots are very rare, since the drive against them is too great to enable them to reach appreciable frequency. The mutation rate towards such hotspots would need to be unfeasibly large for them to be likely to be observed. In our model, hotspots evolve essentially as deleterious mutations within a population. Larger populations harbor more diversity but strongly resist the influx of negative mutations. If hotspot-promoting alleles do arise that cause a hotspot locally, then the pattern of hotspot heats should be very strongly biased towards weak hotspots.

However, the true distribution of inferred heats of hotspots in human populations [2,3] shows a much less dramatic skew towards weak hotspots (although current approaches probably have greater power to infer the presence of hotter hotspots). For example, the presence of the hot human DNA3 hotspot is incompatible with drive acting against new hotspots (Figure 3). In other words, human populations do face a kind of hotspot paradox—how can we explain the existence of our hotter hotspots? To resolve this question, we must examine alternative hypotheses of how hotspot alleles behave. We consider two possible explanations: first, selection for recombination hotspot alleles to ensure correct segregation, and second, that drive does not act against hotspot alleles immediately upon introduction into the population.

### Selection for Correct Segregation

To ensure correct chromosomal segregation in meiosis (in humans and other organisms), at least one crossover per chromosome arm occurs [32]. Further, it has been observed [33] that women with a higher crossover rate have more offspring on average. Hotspot evolution could therefore be influenced by direct selection for higher recombination rates. A simple selection model suggests itself; the offspring is inviable unless at least one *crossover* occurs within a certain region, e.g., a chromosome arm.

We now consider the evolution of a hotspot when crossover within a certain region must occur for the offspring to be viable. Let *r*_H_, *x,* and *p* be defined as before, and suppose now that the probability of a crossover event elsewhere within the region is *w,* independently of whether crossover occurs at the hotspot in question (i.e., no interference). Suppose also that with probability *q* a DSB at this hotspot results in crossover. As before, this model is equivalent to a model of selection with no dominance, and the population scaled drive coefficient against the hotspot allele under this new model is

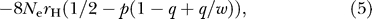
which can be compared to Equation 1. Therefore the effect of selection for recombination is equivalent to increasing the probability of transmission of the hotspot allele, as mathematically the parameter *p* can be replaced by an effective *p*′ = *p*(1 − *q* + *q*/*w*). If the probability of crossover elsewhere in the region (*w*) is small enough, then the hotspot allele is actually (selectively) favored, i.e., *p*′ > 1/2. However, if crossing over is very likely elsewhere in the region, *w* close to 1, the positive effect of crossover in the hotspot becomes negligible. Models of selection for correct segregation under a weaker scenario in which selection acts in only one sex, and an extreme case in which the fitness is proportional to the number of crossovers, are discussed in Text S1 section 7. In all cases, the selection for correct segregation will only have a strong effect at an individual hotspot if recombination elsewhere in the region is unlikely (small *w*) and DSBs within the hotspot often results in crossovers (*q* close to 1).

For most human chromosome arms, *w* ≥ 1/2 for both males and females [33]. In a number of hotspots studied [24], gene conversion is much more likely than crossing over, having at most a probability *q* = 1/5 that DSBs result in crossover. These two estimated quantities (*w* and *q*) give an effective *p*′ of ≤ 1.2*p,* thus *p*′ is still much less than 1/2, and so the impact of such selection at hotspots may well be slight. This model provides a crude upper limit on the selection for correct segregation, as it does not account for the fact that mis-segregated gametes often abort early in pregnancy [32], and so individuals can mate again to produce a viable offspring, reducing the effect of a low recombination rate on an individual's fitness. Thus, it is unlikely that the drive against hotspot alleles could be negated by selection for correct segregation. However, these results suggest that there could be preferential survival of hotspots where DSBs resolve mainly into crossover events; for example the NID1 hotspot where *q* ≈ 4/5 [15].

Looking at the broader chromosomal scale, we can see that selection for correct segregation would have an overall influence on the makeup of hotspots within chromosomes, because the selection will affect the balance between the loss and gain of hotspots. Smaller chromosomes will, all other things being equal, tend to have a lower total mutation rate introducing hotspots. Thus, they will achieve the necessary higher recombination rates by having more hotspots (because negative drive would occur only at higher hotspot densities) and hotter hotspots (because the strength of the drive would be effectively reduced).

### Hotspots That Do Not Initially Experience Drive

An alternative explanation of the presence of intense hotspots is that there might not in fact be mechanistic drive against some newly arisen hotspots. There are a number of plausible reasons why newly arisen hotspot alleles might not experience drive. First, a change to the DNA sequence at one location may introduce a hotspot at some distant location. The allele causing the hotspot would therefore be unaffected by the drive at the hotspot that it introduces. Second, hotspots appear to compete for a finite amount of recombination with other surrounding hotspots [34–37]. As a result, hotspots that are intense in the present day might have been relatively cool in the past. Hence, the allele that causes the hotspot might have originally experienced little drive. Third, evolution of the recombination machinery could cause whole classes of hotspots to be turned on or off simultaneously; e.g., if the motif underlying hotspot activity is changed. Hotspot alleles would spread neutrally before activation of the new motif, and only subsequently be subject to biased gene conversion. Fourth, there is evidence from an experiment in yeast that a hotspot allele in heterozygotes can stimulate DSBs on both chromosomes equally [38]. If alleles that introduce hotspots have such a property, or if, conversely, such alleles do not stimulate DSBs in heterozygotes, they will not experience drive.

A common feature of all these models of hotspot genesis is that the hotspot allele is initially shielded from the drive and thus is neutral. Subsequently, when disrupting mutations arise within an established hotspot, they would still benefit from drive in their favor. To simplify the modeling of such cases, we concentrate only on the number of hotspots currently fixed in the population. More generally, this result will provide intuition as to the expected number of hotspots at high frequency in the population.

We once again assume that the mutation rates are μ_H_ towards alleles that generate a hotspot somewhere in a region, and μ_D_ towards alleles that disrupt a given existing hotspot, where μ_H_ and μ_D_ are now both assumed small. This allows us to approximate our model as a model with two stages in the evolution of a hotspot. During the first stage, before any disrupting alleles have arisen, the newly introduced neutral hotspot allele drifts to either loss or fixation in the population. If the hotspot allele reaches fixation, in the second stage, disrupting alleles arise and lead to the removal of the hotspot if one of them fixes in the population.

The properties of this model are dictated by the rate at which hotspot alleles arise and fix in the population and the rate at which fixed hotspots are removed from the population by disrupting alleles. The rate at which neutral hotspot alleles fix in the population is given by μ_H_. Similarly, hotspots are removed from the population at rate 8μ_D_ *N*_e_ *r*_H_(1/2 − *p*)/(1 − exp(−8*N*_e_ *r*_H_(1/2 − *p*))), which is the rate at which disrupting alleles arise and get fixed in the population (see Text S1 section 8). The expected number of currently fixed hotspots in the region is given by the ratio of the rate at which hotspot alleles arise and fix to the rate at which each given hotspot is lost by fixation of a disrupting allele (see Text S1 section 8 for more details). Thus, the expected number of hotspots of heat *r*_H_ fixed in the population is

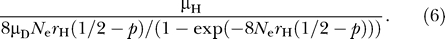
For strong biased transmission in favor of a hotspot-disrupting allele, this equation behaves as μ_H_/(8μ_D_ *N*_e_ *r*_H_(1/2 − *p*)). Thus, the number of fixed hotspots of heat *r*_H_ decays linearly with both *N*_e_ and *r*_H_. Importantly, this drop-off with large *r*_H_ is far less severe than in the case in which hotspots experience drive against them from their introduction into the population, where the drop-off with heat (as can be seen in Figure 3) is approximately exponential. Among possibilities we have explored here, the model in which hotspot alleles do not initially experience drive offers by far the most convincing explanation of why we see hot hotspots in the human genome.

### Do Segregating Hotspot Alleles Leave a Signature in Population Genetic Data?

Hotspots influence patterns of LD, and so it is also of interest to attempt to understand in detail the relationship between hotspots and these patterns, in the case in which hotspot allele frequencies have varied through time. In particular, knowledge of which alleles influence hotspot activity would add to our understanding of hotspots, and an ability to detect such alleles through LD patterns represents an indirect path to finding such alleles.

Thus far, we have considered models of alleles that influence the heat of hotspots forward in time; in order to understand the effect of such alleles on current day patterns of diversity, we must consider the ancestry of the sample backward in time. This leads naturally to the use of a coalescent-with-recombination model [39] to describe that ancestry; the model is similar in many ways to the standard coalescent-with-recombination model, but complicated by the fact that the process of recombination must be modified. The process differs from the normal coalescent model in three important respects. First, the two different allelic backgrounds, A and B, where A is the hotspot-allele, will recombine at different rates backward in time. Second, the parental allelic types of a recombinant chromosome are not random draws from the population. Third, there is an asymmetric distribution of material contributed to the offspring by the parental chromosomes. For example, when gene conversion occurs, there is an asymmetry in which parent contributes the majority of the material.

The derivation of the process is somewhat involved, and so we restrict ourselves to discussing the likely effects of driven alleles at hotspots. A full description of the scheme is discussed in Text S1 sections 9–12, and an algorithm for simulating the coalescent process of a region surrounding a segregating hotspot allele is given in Text S1 section 13. Hellenthal et al. (2006) [40] have independently studied the first point outlined above, but did not formally develop the latter two points.

One interesting case occurs when an allele experiences perfect biased gene conversion (i.e., *p* ≈ 0) in a hot hotspot (*r*_A_ >> *r*_B_). Inspection of the model described in Text S1 section 9–12 shows that ancestral lineages recombine at the same rate regardless of whether they are of type A or B, despite the two backgrounds having very different rates forward in time. Initially, this result perhaps seems counter-intuitive; we might expect the A allele haplotype to recombine far more often than the B haplotype, backward in time. However, the A allele is frequently *not* transmitted when recombination occurs. Thus, the fact that an A allele has been transmitted to the present day implies that it has been involved in few historical recombinations. Therefore, we do not expect type A and B haplotypes to have particularly different patterns of LD; both backgrounds should show similar signals of the hotspot.

Further inspection of the model, the second point in particular, shows that the A and the B haplotype backgrounds will look quite similar, reducing the chances of identifying alleles that affect hotspots by haplotypic patterns. When a recombination occurs in an individual with a B allele, it is likely that the individual's other haplotype has an A allele (as B homozygotes have a much reduced hotspot compared to AB heterozygotes). The transmitted allele from this individual will be the B allele, but the transmitted haplotype will be a mixture of the A and B haplotypes. If the gene conversion or crossing-over rate is quite high, many B chromosomes will be descended from ancestors on the A background in the recent past. Thus, the difference between the A and B haplotypes is eroded.

There is still some hope of detecting polymorphic hotspots from patterns of LD. In general, the rates of crossover and gene conversion will change backwards in time as the frequency of the allele varies. Eventually, when we reach a time before the introduction of the hotspot allele into the population, crossing over in the region will be much reduced. For recently arisen hotspot alleles, there may be some information about this change, due to their relatively recent introduction into the population, but this could be confounded by the reduced power to observe such hotspots. However, the possibility of such a signal remains an area worthy of future exploration.

## Discussion

Several lines of evidence now support the idea that human hotspots vary in intensity and location through evolutionary time. First, segregating mutations near to the center of the DNA2 and NID1 hotspots affect the recombination rate [14,15]. Second, hotspots appear to evolve quickly over evolutionary timescales, with human hotspots typically not conserved in comparisons with our nearest relative, the chimpanzee [7–8]. Finally, examination of a group of hotspots in a 200-kb region of the human genome around minisatellite MS32 [9] revealed strong differences between recombination rates estimated using population genetic data, and male recombination rates estimated using direct sperm typing.

Biased gene conversion at hotspots preferentially fixing alleles that disrupt hotspot activity offers a plausible explanation for the above findings. Our aim here was to consider a modeling framework for such biased conversion, enabling us to explore the implications of such a model. Using the model, we sought first to consider whether a model of biased gene conversion could explain the observed rapid evolution of hotspots, while remaining consistent with the large range of hotspot intensities in humans. Beyond this, the use of such a model enables us to make predictions regarding the signature of this phenomenon, both on a broad genomic scale across many recombination hotspots, and at the level of detecting whether an individual hotspot has recently been influenced by a segregating allele, through consideration of the genealogical process under biased gene conversion.

Estimates of human parameter values suggest that drive is a sufficiently powerful genetic force to ensure that most hotter hotspots will not be shared with chimpanzees, provided that a sufficient number of sites close to the hotspot exist where a mutation can reduce the hotspot heat. Therefore biased gene conversion could create the observed lack of sharing among species. It is worth noting, however, that in general the probability of sharing among species is strongly influenced by the strength of drive, which increases with the heat of a hotspot. Therefore, intense hotspots are much more vulnerable to extinction through the process of biased gene conversion, other things being equal. A survey in humans and chimpanzees of a number of relatively cold hotspots, perhaps in cold regions where they would more readily be detectable from LD patterns, might well show some proportion of conserved hotspots, if driven alleles are the principal cause of hotspot extinction.

In view of the ability of drive to destroy hotspots, a key question is whether one ought to see hotspots at all if drive is acting [11,12]. Provided hotspots can arise in the population, there will be some stationary distribution of their number (and frequencies, considering a hotspot as a potentially segregating allele within the population) for different intensities of hotspots, even if there is biased gene conversion against any new hotspot allele entering the population. Interestingly, this distribution depends strongly on the effective population size *N*_e_. For larger population sizes, if hotspot alleles must arise against drive, then there is a strong bias towards hotspots of lower heat, with powerful suppression of hotter hotspots. Perhaps the “hotspot paradox” issue [11] that we must address is not how hotspots persist in the face of drive but why there are very hot hotspots within the genome.

We considered two possible resolutions of this paradox; direct selection on the chromosomal scale for higher levels of recombination, and a lack of drive affecting newly arisen hotspot alleles. Direct selection for higher rates was proposed recently by Kong et al. (2004) [33] for maternal recombination. Further, some such selection seems highly credible, given the requirement for around one crossover per chromosome arm in humans and many other organisms. However, for human levels of recombination, such selection seems unlikely to have a significant effect at individual hotspots, except perhaps in a minority of extreme cases such as within the PAR1 pseudoautosomal region, where a large male recombination distance (50 cM) is compressed into a very short stretch of the genome (3 Mb).

The second possibility that we considered as an explanation of the abundance of hot hotspots is that newly arisen hotspot alleles do not compete against biased gene conversion favoring the ancestral, non-hotspot type (this drive would be comparable to the positive drive affecting an allele suppressing the hotspot). In biological terms, drive favoring the ancestral type implies that a hotspot-stimulating mutation creates a hotspot very locally in *cis*. This assumption may well be inaccurate; hotspot-causing mutations might act remotely and so not suffer from such drive. Under such departures, hotspots of high heat are much more frequently fixed within the population, although these then typically survive for less time than weaker hotspots.

A similar dynamic will also be achieved if competition exists between local hotspots for some finite amount of recombination [34–37,41]. This competition between hotspots could initially suppress the heat of new hotspots, reducing the drive against the alleles causing them, and thus allowing some of these alleles to rise in frequency due to genetic drift. When hotspots surrounding a new hotspot are removed or cooled by disrupting alleles, the new hotspot allele would increase in intensity and could already have drifted to high frequency in the population. The hotspot could itself then be removed from the population, at a rate proportional to its new heat, by disrupting alleles. Finally, evolution of the hotspot machinery itself could have the most dramatic effect of all, by turning on or off many hotspots across the genome simultaneously. Any of these scenarios, in which hotspots can arise without facing meiotic drive against them but where such drive can cause future extinction, seem broadly consistent with present observations.

Although selection for high rates does not in general seem to explain *very* hot hotspots, it could be an important force in regulating overall recombination rates. Selection for a rate giving at least one crossover event per chromosome arm would essentially multiply (downweight) the drive for any allele disrupting a hotspot. On shorter chromosome arms, a selectively favored higher rate would naturally be achieved by a combination of both more, and, on average, slightly hotter, recombination hotspots. This is consistent with observations in Saccharomyces cerevisiae, in which shorter chromosomes have both a significantly higher density of hotspots and hotter hotspots [42].

The various ways in which hotspot intensities can change might well be highly variable and are, as yet, poorly understood. For example, Tiemann-Boege et al. (2006) [41] find a hotspot that varies in position across men, while Neumann and Jeffreys (2006) [43] finds two adjacent hotspots in which local sequence does not appear to determine activity. The different ways in which hotspots can vary suggests that the evolution of hotspots is likely to be complex—a fact further suggested by our model-based analysis—and, although biased gene conversion is likely to be a key component in the evolution of fine scale recombination rates, non-local factors are probably equally influential.

Finally, we turn to the effect of segregating hotspot alleles on population diversity patterns. The effect is strong, since hotspot activity varies through time, meaning hotspots might appear much colder or hotter from patterns in the data than their present day prevalence and heat would suggest. Inferring this signal directly from population genetic data is far more difficult, since rates on the two allelic backgrounds back in time remain similar to one another, eroding differentiation. In particular, we need to be able to reconstruct ancestral background patterns and to observe ancient crossover events, which will be problematic in practice.

## Materials and Methods

The methods used are included in the Results section and in Text S1.

## Supporting Information

Text S1 Supplementary Material (121 KB PDF)

## Abbreviations

DSB: double-strand break
LD: linkage disequilibrium
