# Supplementary material for: Live Hot, Die Young: Transmission Distortion in Recombination Hotspots
Source: PLoS Genet. 2007 Mar 9;3(3):e35. doi: 10.1371/journal.pgen.0030035 (PMC1817654; doi:10.1371/journal.pgen.0030035)
Supplement: Supplementary file 1 [file pgen.0030035.sd001.pdf]

Supplementary material

Live hot, die young: transmission distortion in recombination hotspots

Graham Coop[†] and Simon R. Myers[*]

[†] Department of Human Genetics, University of Chicago,

920 E 58th Street CLSC 505, Chicago, Il 60637

[*]The Broad Institute of Harvard and Massachusetts Institute of Technology,

1 Kendall Square, Cambridge, Massachusetts 02139, USA

Running header: Transmission distortion in recombination hotspots

Both authors contributed equally to the work

SOM 1: Modeling the frequency of an allele experiencing drive

Suppose that the current frequency of the hotspot allele is x, in a diploid population with effective size N_e. The expected frequency of the A allele in the next generation is

$$\mathbf{E}(x') = (1-\mu_D)\Big[x^2 + 2x(1-x)\big\{1/2(1-r_A-r_B) + r_A p + r_B(1-p)\big\}\Big]. \tag{1}$$

Assuming that r_A, r_B and μ_D are small (such that $r_A\mu_D \approx 0$ and $r_B\mu_D \approx 0$) and writing $r_A - r_B = r_H$ then the expected change in frequency, $\mathbf{E}(x') - x$, is

$$-2r_H(1/2-p)x(1-x) - \mu_D x. \tag{2}$$

where the first term is the change due to the drive in heterozygotes and the second term to mutation out of the hotspot allele. For a large effective population size N_e, this model is well approximated as a simple diffusion process (scaling time in units of $2N_e$, as N_e tends to infinity, see Ewens [2] for an introduction) with diffusion parameter $x(1-x)$ and drift parameter

$$-4N_e r_H(1/2-p)x(1-x) - 2N_e\mu_D x, \tag{3}$$

which is merely equation (2) scaled by $2N_e$.

SOM 2: Obtaining human parameters

In order to obtain the estimated human drive parameters shown in table 1 of the main text, we based calculations on (3) with $\mu_D \approx 0$, and $N_e = 10,000$. For each hotspot, the intensity of crossover in males has previously been estimated [7] (DNA1–3), [10] (NID1) and [14] (SHOX). At two of these hotspots, DNA2 [9] and NID1 [10] a marker was identified for which one of the two types disrupted hotspot activity, and estimates were obtained of both the crossover rate r_h, and the probability p' of transmission of this marker in recombinants, for heterozygotes. We assumed the same rates in females as in males throughout.

It is easy to show that the population scaled drive parameter (3) is given by

$$8N_e r_H(1/2-p) = 8N_e r_h(1/2-p')$$

and we substituted in $p' = 0.24$ for DNA2 and $p' = 0.26$ for NID1 to obtain the strength of drive for these hotspots. To estimate crossover-based drive for the other hotspots, we used the same p' as for DNA2 and a crossover rate as shown in the table.

To estimate the gene-conversion based drive we modified the above to include the fact that at a chosen marker, under the DSB model we can only observe gene conversion occurring if the initiating chromosome is transmitted to the offspring (with probability 1/2). If r_h is now the observed gene conversion rate, and p' is the proportion of gene conversion products carrying the disrupting allele, and r_H is now the rate (in heterozygotes) of DSBs that result in gene conversion, the contribution of simple gene conversion to the scaled drive is

$$8N_e r_H(1/2 - p) = 16N_e r_h(1/2 - p').$$

Finally, to estimate the gene-conversion contribution we used direct estimates of $r_h = 1.3 \times 10^{-4}$ and $p' = 0.29$ for NID1 [10], and for the other hotspots we used the same value of p', estimates of $r_h = 1.3 - 3.4 \times 10^{-3}$ for the most-frequently converted marker at hotspot DNA3 [8], and then assumed the same ratio of crossover to conversion at the remaining four hotspots as for DNA3. The estimates produced are therefore very approximate, since recombination rates in females are unknown, there is uncertainty in the rate estimates, and p' and the assumed gene conversion rates are extrapolated from other hotspots in most cases. The SHOX hotspot is in the psuedoautosomal region of the Y chromosome, which is subject to an obligate crossover in males, and likely to be much less active in females. We have assumed that the SHOX hotspot is inactive in females, and so the sex-averaged recombination rate for the SHOX hotspot is half that observed in males. Further, we have assumed implicitly here that in heterozygotes for the disrupting mutation the recombination rate would equal the current population rate; an additive model of intensity might instead suggest a smaller recombination rate in heterozygotes, perhaps as little as half the population rate, and in this case the drive parameter should be reduced by this factor.

SOM 3: Probability that a hotspot survives uncooled from ancestral populations

Using the parameterisation defined in the main text and standard diffusion theory, an allele that arises via a single mutation (so at frequency $1/2N$), and lowers the rate of DSB initiation by r_H, has an approximate probability

$$2gN_e/\big(N(1 - \exp(-4N_e g))\big) \tag{4}$$

of achieving fixation (and thereby weakening, or removing, the hotspot). Mutations that disrupt the hotspot occur at rate $2N\mu_D$, therefore the rate at which disrupting alleles arise

and fix in the population is approximately $2N\mu_D\times$ eq(4). As r_H becomes large, this death rate increases approximately linearly with hotspot intensity, the mutation rate (or the number of sites able to disrupt the hotspot through their mutation) and the effective population size N_e. Neglecting the possibility of a currently segregating disrupting allele, or of several disruptive mutations co-segregating within the population at one time, the probability of survival of a hotspot in both species from the ancestral population to the current day is approximated by

$$\exp\left\{-2N_e\mu_D\frac{2g}{1-\exp(-4N_eg)}\times 2T\right\}. \tag{5}$$

This approximation is commonly used for selected alleles [11] and we found it to provide an excellent approximation, for the parameter values used in this paper, to the probability obtained via direct simulation using a Wright-Fisher simulation (results not shown).

SOM 4: The frequency spectrum of hotspot alleles

Suppose within a region of DNA sequence, the rate of introduction of hotspot alleles is μ_H and the rate of introduction of a mutation killing any such hotspot is μ_D (note that μ_D is the rate per hotspot, while μ_H is the rate of hotspot introduction in the region). Then the expected number of hotspots with population frequency in a small frequency interval $[x, x+dx)$ is given by

$$4N\mu_H\frac{\exp\big(-4N_egx\big)}{x(1-x)^{1-4N_e\mu_D}}dx. \tag{6}$$

This equation is obtained by modifying the expected number of selected alleles with frequency x in a model with one way mutation [13], to reflect the hotspot parameters. Note that the above expression implies that exactly as for polymorphic sites there could potentially be a large number of hotspot alleles segregating at low frequency in the population. We assume that the effective population size is the census population size here in order to simplify our results (this assumption is common and in our case has little effect as it scales only the mutation rates).

SOM 5: Simulations to investigate demography

We sought to consider whether human population size changes might strongly influence the evolution of hotspots, and in particular if such events could result in more active hotspots reaching high frequency in the population. Human populations are likely to have experienced both recent expansion and bottleneck events. Population growth from some ancestral size will increase the efficacy of drive against hotspots relative to the ancestral size, and so result in fewer hot hotspots reaching fixation, so we did not consider this possibility in detail. The intensities we use to illustrate the likely strength of drive for human hotspots were all estimated in European populations, which are highly likely to have experienced a bottleneck [18], so it is important to consider the effect of such events. We therefore examined whether hotspots of the observed intensities of each of DNA1, DNA2, DNA3 and NID1 were likely to have arisen against biased gene conversion under realistic population scenarios incorporating such a bottleneck. Voight et al. [18] provided a range of bottleneck intensities and durations for a European Italian population consistent with multiple aspects of genetic data, and the historical relationships between populations. We separately simulated the evolution of hotspots with each drive parameter $2r_H(1/2 - p)$, estimated for DNA1, DNA2, DNA3 and NID1, for two different demography models, representing the extremes consistent with the European data of Voight et al. [18]). The first demographic model has constant population size 10,659 until a severe bottleneck beginning 40,000 years (1600 generations of 25 years) ago, and reducing the population size 10-fold for 400 generations, before a return to the original population size, whilst the second has a milder bottleneck beginning at the same time but reducing the population size 2.5-fold for 1600 generations. Although neither of these models is likely to be perfect, we believe they do provide a sense of the impact of plausible population size changes, at least in the European group, on hotspot intensity distributions. For each model, we set the mutation rate away from hotspots to be low, and 25 times the mutation rate towards hotspots (10^{-8}), as in Figure 3. (The effect of altering these mutation rates is chiefly to alter the absolute number of hotspots in the population, rather than the relative counts of hotspots as a function of heat.) For each of the four intensities and the two

demographic models, we simulated 25,000,000 generations of evolution of the population, to ensure convergence to stationarity, 20,000 times and measured the proportion of simulations that a hotspot at frequency above 50% in the population was observed in the present day (in fact we increased the efficiency of simulation by reducing all times and population sizes by a factor of 10, while increasing the recombination and mutation parameters by factors of 10, to achieve the same population dynamics but at 100-fold reduced computational cost). The results, shown graphically in Figure 3, indicated at most a weak impact of these bottlenecks on the hotspot distribution relative to constant population size models, and in particular we never observed hotspots as intense as either NID1 or DNA3 in our simulations. We also performed similar simulations for a constant population size of 10,000 - the results (not shown) showed excellent agreement with the theoretical predictions plotted on Figure 3.

SOM 6: Upper bound on the number of hotspot alleles with apparent population heat r_H

It is reasonable to assume that the rate of recombination inferred by an LD based approach for a segregating hotspot can be thought of crudely representing a time average of the mean heat of the hotspot across individuals in the population. Thus an intense hotspot that has always been at low frequency would be inferred to be a less intense hotspot by LD based methods. To capture in part this effect we construct an upper bound on the "apparent" intensity of a segregating very intense hotspot allele, and consider the spectrum of population intensities of hotspots using our upper bound.

Consider a hotspot allele (with recombination rate r_H) presently at frequency x in the population. Imagining that the hotspot allele was held at this frequency through time, it would appear to have a rate of recombination $r_H x$ in the population (i.e. the rate inferred by LD based approaches). However if this hotspot allele has a high rate of recombination and hence strong drive against it, in the past the allele will typcially have had frequency below

x (its current frequency will be the highest frequency achieved, as for intense hotspots the frequency will decrease roughly deterministically backward in time). Thus $r_H x$ is normally an upper bound on the population estimated rate of an intense hotspot allele with current frequency x. Assuming this upper bound, each hotspot of heat r_H/x which are currently at a frequency x in the population has an apparent heat r'_H. The density of hotspots with heat r_H and at frequency x is:

$$4N_e\mu_H \frac{\exp\big(-8N_e r_H(1/2-p)x\big)}{x(1-x)^{1-4N_e\mu_D}} dr_H dx \tag{7}$$

and we can transform to units of apparent heat r'_H using the relationship $r'_H = r_H x$) to give density of hotspots of inferred heat r'_H and at frequency x:

$$4N_e\mu_H \frac{\exp\big(-8N_e r'_H(1/2-p)\big)}{x^2(1-x)^{1-4N_e\mu_D}} dr'_H dx. \tag{8}$$

Finally, to find the total density of hotspots with an upper bounded heat of r'_H we integrate over the frequency of the hotspot allele

$$4N_e\mu_H \exp\big(-8N_e r'_H(1/2-p)\big)\int_\alpha^1 \frac{1}{x^2(1-x)^{1-4N_e\mu_D}} dx \tag{9}$$

where the lower limit on the integral, $\alpha \geq 1/2N$, is the minimum frequency a hotspot needs to achieve in practice to have apparent intensity r'_H (and in particular if any new hotspots have maximal intensity c, $\alpha \geq r'_H/c$). Thus for large hotspot intensities the number of hotspots estimated to have heat r'_H decreases at least exponentially.

SOM 7: Some alternative models of selection for recombination due to correct segregation

It is straightforward to show that under more general models of selection for crossing over, an expression of the same form as equation (4) of the main text holds for the drive parameter, where we must merely substitute w' for w to obtain the appropriate (modified) scaled drive

parameter:

$$-8N_e r_H(1/2 - p(1 - q + q/w')). \tag{10}$$

For example, our model of selection for recombination may be more appropriate for female meiosis, where a far higher percentage of gametes with an incorrect number of chromosomes are allowed to proceed through meiosis [6] and thus perhaps lower reproductive success. In contrast, a relatively low recombination rate in male meiosis, although lowering the proportion of viable sperm, might have somewhat less of an effect on male fertility. In fact, the qualitative effect of such selection is similar whether recombination fraction in males affects fertility or not. In this case, where the recombination rate only influences fertility in females, we find $w' = 2w/(1 + w)$, where w is the probability of crossing over in a region in females.

Similarly, under an even more dramatic model of selection for recombination where fitness is proportional to the number of crossover events C, and if this number of events has finite expectation $E(C)$ then $w' = (1 + 1/E(C))^{-1}$ (corresponding to more favourable conditions for a hotspot allele compared to the previous cases, by Jensen's inequality).

SOM 8: Expected number of fixed hotspot alleles

A slightly more general form of the expected number of fixed hotspot alleles, equation (6) of the main text, can be derived. We assume that the hotspot allele increases the probability of a DSB initiation by r_H^*, and that any locally disrupting mutation reduces this probability of initiation by r_H. In a heterozygote for the ancestral and hotspot allele the probability that the initiating allele is transmitted is p^*. Similarly, in a heterozygote for the disrupting and hotspot allele the probability that the initiating allele is transmitted is p.

Hotspot alleles, as before, are introduced at rate $2N_e\mu_H$ and reach fixation with the probability given by equation (4) modified to reflect that the drive $8N_e r_H^*(1/2 - p^*)$ acts against the allele instead of for it. Note that for $p^* = 1/2$ this probability converges to $1/2N_e$, since the allele experiences no drive and therefore acts as a neutral allele. The

probability of a disrupting allele reaching fixation is given by equation (4). The expected number of currently fixed hotspots in the region is then simply the ratio of the rate at which hotspots arise and fix to the rate at which each given hotspot is lost by fixation of a disrupting allele. The distribution of the number of fixed hotspots is Poisson (through a simple application of queuing theory), with expectation

$$\frac{\mu_H r_H^*(1/2-p^*)/\big(\exp(8N_e r_H^*(1/2-p^*)-1)\big)}{\mu_D r_H(1/2-p)/\big(1-\exp(-8N_e r_H(1/2-p))\big)}. \tag{11}$$

In addition to mutations that locally disrupt the hotspot, a hotspot may also be disrupted by alleles at remote loci that do not benefit from the drive. However, unless the rate at which these arise is much greater than the rate at which locally disrupting alleles arise they are unlikely to play a strong role in the evolution of hotspots. They may however, play a part in the evolution of relatively cold hotspots.

SOM 9: The Coalescent process with biased transmission

We suppose that the DSB initiation process is additive, i.e. the rate of initiation in AA homozygotes is $2r_A$ and that the rate of initiation in BB homozygotes is $2r_B$ (the fully general case is easily treated in the same way as presented here). Table 1 shows the joint probability of the offspring allelic type (denoted by O) and the DSB events that could have occurred in the parents (given that the frequency of the A allele in the parental generation is x). Using these joint probabilities we can construct the conditional probabilities of various events. Note that the first and second row of the table sum approximately x and $1-x$ respectively (as the frequency of a particular allelic type, $P(0)$, alters little between two generations).

O	No DSB	DSB in homozygote	DSB in heterozygote
A	$(1-r_A-r_B)x(1-x)+(1-2r_A)x^2$	$2r_Ax^2$	$2(r_Ap+r_B(1-p))x(1-x)$
B	$(1-r_A-r_B)x(1-x)+(1-2r_B)(1-x)^2$	$2r_B(1-x)^2$	$2(r_Bp+r_A(1-p))x(1-x)$

Table 1: The joint probability of the offspring type O and events in the parent, given that the frequency of the A allele in the parental generation is x.

SOM 10: Background-specific rate of recombination

In the normal ancestral process both backgrounds A and B recombine at the same rate. Consideration must be given now to the probability that a DSB happens in the previous generation, on a haplotype with a particular allele, A or B; this will be influenced by not just the differing probabilities of initiation, but also by the very fact that this allele was transmitted through the recombination event. If the frequency of the A allele in the previous generation is x, then conditional on the offspring type in the current generation, O, being A, the probability of a DSB in the previous generation is

$$\begin{aligned} P(\text{DSB}|O=A) &= P(\text{DSB},O=A)/P(O=A) \\ &= \frac{1}{x}\big\{2x^2r_A+2x(1-x)(r_Ap+r_B(1-p))+(1-x)^2\times 0\big\} \\ &= 2\big\{xr_A+(1-x)(r_Ap+r_B(1-p))\big\}, \end{aligned} \quad (12)$$

and by a similar argument

$$P(\text{DSB}\mid O=B)=2\big\{(1-x)r_B+x(r_A(1-p)+r_Bp)\big\}. \quad (13)$$

Note that although this formula strictly applies to the probability of DSBs on each background, it is directly applicable to the probability of crossover, if we instead let r_A and r_B be the crossover rates for the two alleles and p be the transmission probability of the initiating allele conditional on a crossover in a heterozygote. For example, in the case of the NID1 hotspot the allele disrupting the hotspot has the same conversion transmission properties (i.e. the same p) regardless of whether or not the conversion is accompanied by crossover, and so $P(\text{crossover}\mid O=A)=qP(DSB\mid O=A)$ where q is the fraction of DSBs that

result in crossover (and likewise for $P(\text{crossover} \mid O = B)$). Similarly, we can directly apply it to the case where gene conversion is not accompanied by crossover; this is useful in full consideration of the ancestry (see the next section).

When the DSB process is completely biased against the initiating allele, i.e. $p = 0$, then from equations (12) and (13) both A and B lineages recombine at rate $2[xr_A + (1 - x)r_B]$. This at first seems counterintuitive, since forward in time more recombination occurs on the hotspot allelic background. However, the fact that the allele was transmitted acts in such a way as to counter this effect. The argument for equal rates can be phrased as follows for $p = 0$. The offspring of recombinants are always the non-initiating allele, and the a type of the non-initiating allele is simply drawn from the population frequency at random. Following lineages back in time, we are constantly following non-initiating types through any recombination events encountered. Further, since at any such recombination these are drawn at random from the population, the type of a lineage gives no information about the recombination rate on that lineage.

In contrast, if $p = 1$ the initiating allele is *always* passed on, and in this case equations (12) and (13) give $P(\text{DSB} \mid O = A) = 2r_A$ and $P(\text{DSB} \mid O = B) = 2r_B$, and so there can be a distinct difference between the rates of recombination on either background. In a more general setting where initiation rates are nonadditive (e.g. when there is no dosage effect on the rate of DSB initiation, as found at a hotspot in mice [20], and equally consistent with observations in humans at the DNA2 hotspot [9]) a similar simple formula can be obtained, and in this setting it is even possible for the B background to have a higher historical rate than the A background!

SOM 11: Background-specific choice of parental alleles

Conditional on a DSB on a particular allelic background, A or B, one of the parental allelic types must be the same as the offspring (we view the allelic type, A or B as determined essentially at a single locus) as it has transmitted this allelic type information. We need to calculate the conditional probability of the allelic type of the other parental chromosome being either A or B (this is important since both parents will contribute some genetic material to the offspring chromosome). When considering an allele which does not affect the probability of transmission or the rate of recombination, the other parental allele is picked at random from the current population frequency of the allele [5]. However in this case the choice of parental allele is influenced by the fact that a recombination has happened, as forward in time the probability of a DSB is altered by whether the parent is AA or AB. We now turn our attention to the choice of parental allelic types that formed the DSB resulting in the offspring chromosome. One parental chromosome determines the offspring allele type, and in SOM 10 we consider what other material is copied this chromosome. Given a DSB in the previous generation producing an offspring type A, one parental chromosome is also of type A (transmitting this allelic type to the offspring chromosome) and the other can be A or B. The unknown parental genotype is denoted by G, where $G = AA$ or $G = AB$, and the offspring type by $O = A$. The probability that both parental chromosomes that formed the DSB are of type A given an offspring of type A can be written as

$$\begin{aligned}
P(G = AA \mid \mathrm{DSB}, O = A) &= \frac{P(G = AA, \mathrm{DSB}, O = A)}{P(G = AA, \mathrm{DSB}, O = A) + P(G = AB, \mathrm{DSB}, O = A)} \\
&= \frac{2x^2 r_A}{2x^2 r_A + 2x(1-x)(r_A p + r_B(1-p))} \\
&= \frac{x r_A}{x r_A + (1-x)(r_A p + r_B(1-p))}, \qquad (14)
\end{aligned}$$

and by a similar argument for an offspring of type B

$$P(G = BA \mid \mathrm{DSB}, O = B) = \frac{x(r_A(1-p) + r_B p)}{x(r_A(1-p) + r_B p) + (1-x) r_B}. \qquad (15)$$

Once again, these results may be applied directly to crossing over by considering the parameters r_A and r_B as the probability of crossing over for the two alleles, and p as the

probability of transmission of the initiating allele given a crossover in a heterozygote. Similarly it is applicable to gene conversion events unaccompanied by crossing over, with the corresponding probabilities. In the case of perfect biased transmission against the initiating allele, $p = 0$, then the type of the nontransmitted allele is chosen to be A with probability $xr_A / \big(xr_A + (1-x)r_B\big)$, regardless of the allelic type of offspring chromosome. If $r_A >> r_B$ then this last result shows that the nontransmitted allele is always of type A. This result is clear if we look at the example of the ancestry of a B chromosome; if a type B ancestor to this chromosome was in a BB homozygote then a DSB can not have been initiated, while if this ancestor was in a AB heterozygote the A allele must have been the chromosome that initiated the DSB and so was not passed on. On the other hand, when $p = 1$ then with probability x the nontransmitted allele is of type A. This is the same as randomly drawing the allelic type from the population, which is the correct sampling method when there is no difference in the rate of initiation between the allelic types.

SOM 12: Choice of Parental material

Finally, conditional on occurrence of a DSB and the types of the parents we are required to choose what material is given to the offspring, from each parental chromosome. For a gene conversion event this probability determines which chromosome contributes the majority of the material and which has been copied only for the gene conversion tract; and in the case of a crossing over event, this probability gives which parental chromosome is copied for the material to the left of the DSB and then the other is copied for material to the right.

The region of intense gene conversion associated with the hotspot is small [8]. For simplicity we consider only markers outside this region, as there will be few markers within this region (other than the hotspot locus). We also do not model gene conversion outside of the hotspot. A full treatment would have to model the ancestral gene conversion fragments, for example as described in Wiuf and Hein [19]. We effectively assume that the hotspot has

zero width, but finite heat, which allows us to consider gene conversion within the hotspot that does not result in crossing over as a process that merely switches the background of the ancestral haplotype outside of the hotspot. Conditional on a gene conversion event, we can ignore the parent contributing at most the small gene converted fragment, and need follow only the other parent back. For an offspring of type A with parental genotype AB, we follow the A chromosome with some probability f_A, and otherwise the B chromosome. We assume both chromosomes produced by a gene conversion are equally likely to be passed down to the offspring, and consider the case where initiation takes place on an allele's own strand. The initiating allele is passed down with probability p_{nc} which is half the probability that the gene conversion tract does not include the rate determining locus. This enables calculation of the required probability, and we obtain the following;

$$f_A = \frac{r_A p_{nc} + r_B/2}{r_A p_{nc} + r_B(1 - p_{nc})} \tag{16}$$

Similar formulas can be obtained in more general cases, or in the case that an allelic type initiates recombination on the other strand. In this simple formulation, gene conversion essentially just results in 'migration' between types A and B backward in time, with a bias towards migration to the A type from the B type if $p_{nc} < 1/2$.

Analogously, when a DSB results in crossing over the recombinant has two parents, one of these contributes material to the left of the hotspot, and the other to the right. The contribution of the parental can be chosen at random unless the parent chromosomes are of type A and B, i.e. a heterozygote. In this case the probabilities for the two possibilities; A contributing the left or right half; will not be equal in general, and will depend on the offspring type. Arguing similarly to the gene conversion case above, and again assuming that both products are equally likely to be transmitted, a corresponding expression can again be derived (not shown). This can be written in terms of qr_A, qr_B, p_c, and an additional parameter p_{cl}, the proportion of crossovers whose gene conversion tract lies entirely to the left of the allele-determining locus, where $p_{cl} \leq 2p_c$.

SOM 13: How can we model population genetic data featuring a segregating hotspot allele?

Several computational approaches have recently been developed that utilise population genetic data in order to infer variable historical recombination rates and hotspot locations [3, 12, 15]. These offer a distinct approach, with unique advantages and disadvantages, that complements those of linkage and sperm studies. The rate found by methods using population genetic data is clearly in a sense "averaged" over a great number of generations, implying that the experimentally verified hotspots, such as those in the MHC and Beta Globin regions, all of which have an effect on patterns of LD (see for example Fearnhead et al. [3]), must have been active for considerable periods of time. The possibility of segregating hotspot alleles raise a number of interesting questions for detection of hotspots based on variation data. For example: what power do we have to detect such hotspots; how does the heat we infer relate to the true heat; if a hotspot has gone extinct at some time in the past how much of a signal remains, and can we distinguish it from a currently active hotspot? Here we describe a method to simulate the ancestry of a sample, and hence sample variation data, conditional on a current (or at least recent) segregating hotspot allele, in order to enable future quantitative investigation of these questions. We also briefly consider the implications of the derived backward process.

To explore the effect of a segregating hotspot allele in detail, we can model the underlying genealogy by a modified coalescent process. The rate of DSB initiation and the choice of parental background depend on the frequency of the A and B alleles. As noted before the drive is exactly analogous to genic selection. This means that we need to consider simultaneously, backward in time, the ancestry of the sample and the frequency of the hotspot allele. This can be modeled in a similar way to modeling the genealogy when selection acts at a single site, as described in Hudson and Kaplan [5] (see Nordborg [16] for an introduction). Conditional on the frequency of the hotspot allele through time, the genealogical process

can be modeled as a subdivided population where the frequency of each allele gives the population sizes.

Let crossing over result from a DSB with probability q. When a DSB is initiated, if crossing over does not occur the initiating allele is transmitted with probability p_{nc}, while if crossing over does result, it is transmitted with probability p_c. Combining the results of the previous three sections, the following algorithm describes how to simulate from the process.

- Simulate the frequency $\{X_t\}$ of the hotspot allele in the population backward in time from the present day, to the eventual loss of the A allele, approximating the conditional backward diffusion using a birth and death process as described in Griffiths [4] and implemented in Coop and Griffiths [1] (see SOM 12 for more details).
- Initially set $t = 0$, and sample the initial numbers n_A of type A haplotypes and n_B of type B haplotypes according to the appropriate ascertainment model for sampled sequences.
- At a time t into the past, with current frequency $X_t = x$ of the hotspot allele A in the population, some totals n_A of type A haplotypes and n_B of type B ancestral haplotypes still remain. Then events occur to the $n_A + n_B$ ancestors of the sample at the following instantaneous rates:

    1. Coalescence of two ancestral A haplotypes: $n_A(n_A - 1)/2x$;
    2. Coalescence of two ancestral B haplotypes: $n_B(n_B - 1)/2(1 - x)$;
    3. Mutation of an ancestral haplotype of allelic type B to type A (this is the one way mutation out of A viewed in reverse): $n_B\mu_D x/(1 - x)$;
    4. Gene conversion (not accompanied by crossing over) on one of the A or B ancestral haplotypes, rates $n_A\times$eqn.(12) and $n_B\times$eqn.(13) respectively; using $(1 - q)r_A$, $(1 - q)r_B$ and p_{nc} in place of r_A, r_B and p;

5. Crossing over on one of the A or B ancestral haplotypes, rates $n_A\times$eqn.(12) and $n_B\times$eqn.(13) respectively; using qr_A, qr_B and p_c in place of r_A, r_B and p.

- Allow the above process to continue until all sequences have reached a single ancestor (i.e. $n_A + n_B = 1$)

For gene conversion and crossover events, one parental chromosomes will be the same allelic type as the offspring. The allelic type of the nontransmitted allele must also be chosen, using $(1-q)r_A$, $(1-q)r_B$ and p_{nc} for gene conversion (without crossing over) and qr_A, qr_B and p_c for crossing over, from equation (14) or (15) if the ancestral haplotype is A or B respectively. In addition, the material that the respective parents contribute to the offspring must also be chosen, and the probabilities associated with this choice are described in SOM 10.

As usual, the above algorithm can be adapted to save computational time by only following lineages containing DNA material ancestral to the sample, and at positions which have not yet reached a most recent common ancestor (we must also keep track of the type, A or B, at the hotspot allele on such lineages). We can also easily extend to allow crossing over outside of the hotspot region which is dealt with in the usual way, i.e. by drawing the allelic type of the parental chromosome from the current population frequency of the hotspot allele when the crossing over occurs [5].

SOM 14: Simulating the hotspot allele frequency backward through time

The Moran model, a birth and death process, may be used to approximately model the frequency of the hotspot allele through time. This uses the population scaled drift coefficient

$$\mu(x) = -4N_e r_H(q(1/2 - p_c) + (1-q)(1/2 - p_{nc}))x(1-x) - 2N_e\mu_D x, \qquad (17)$$

which is a version of equation (3) modified to reflect the differing biases in the products of DSBs that are and are not accompanied by crossover. The approximating Moran model has

a population size of N chromosomes; which for computational convenience is less than $2N_e$. If there are currently j $(0 < j < N)$ chromosomes with the hotspot allele in the population, the Moran model has birth rate

$$N(Nx(1-x)+\mu(x))/2 \tag{18}$$

and death rate

$$N(Nx(1-x)-\mu(x))/2 \tag{19}$$

where $x = j/N$. Note that we do not have to condition on eventual loss back in time, since this is guaranteed by the one-way mutation towards the B allele.

References

[1] Coop G, Griffiths RC (2004) Ancestral inference on gene trees under selection. Theor Popul Biol 66: 219–232.

[2] Ewens WJ (2004) Mathematical population genetics, vol. 1. Theoretical Introduction of Mathematical Biology, chap. 5. Applications of diffusion theory. Springer.

[3] Fearnhead P, Harding RM, Schneider JA, Myers S, Donnelly P (2004) Application of coalescent methods to reveal fine-scale rate variation and recombination hotspots. Genetics 167: 2067–2081.

[4] Griffiths RC (2003) The frequency spectrum of a mutation, and its age, in a general diffusion model. Theor Popul Biol 64: 241–251.

[5] Hudson RR, Kaplan NL (1988) The coalescent process in models with selection and recombination. Genetics 120: 831–840.

[6] Hunt PA, Hassold TJ (2002) Sex matters in meiosis. Science 296: 2181–2183.

[7] Jeffreys AJ, Kauppi L, Neumann R (2001) Intensely punctate meiotic recombination in the class II region of the major histocompatibility complex. Nat Genet 29: 217–222.

[8] Jeffreys AJ, May CA (2004) Intense and highly localized gene conversion activity in human meiotic crossover hot spots. Nat Genetics 36: 151–156.

[9] Jeffreys AJ, Neumann R (2002) Reciprocal crossover asymmetry and meiotic drive in a human recombination hot spot. Nat Genetics 31: 267–271.

[10] Jeffreys AJ, Neumann R (2005) Factors influencing recombination frequency and distribution in a human meiotic crossover hotspot. Hum Mol Genet 14: 2277–2287.

[11] Kimura M (1963) On the probability of fixation of mutant genes in a population. Genetics 47: 713–719.

[12] Li N, Stephens M (2003) Modeling linkage disequilibrium and identifying recombination hotspots using single-nucleotide polymorphism data. Genetics 165: 2213–2233.

[13] Maruyama, T (1974) The age of an allele in a finite population. Genet. Res. Camb. 23: 137–143

[14] May CA, Shone AC, Kalaydjieva L, Sajantila A, Jeffreys AJ (2002) Crossover clustering and rapid decay of linkage disequilibrium in the Xp/Yp pseudoautosomal gene SHOX. Nature Genetics 31: 272–275.

[15] McVean GAT, Myers SR, Hunt S, Deloukas P, Bentley DR, et al. (2004) The fine-scale structure of recombination rate variation in the human genome. Science 304: 581–584.

[16] Nordborg M (2001) Coalescent Theory. In: Balding DJ, Bishop M, Cannings C, editors, Handbook of Statistical Genetics, pp. 179–212, Wiley.

[17] Spencer C, Coop G (2004) SelSim: A program to simulate population genetic data with selection and recombination. Bioinformatics 20: 3673–3675.

[18] Voight BF, Adams AM, Frisse LA, Qian Y, Hudson RR, Di Rienzo A. (2005) Interrogating multiple aspects of variation in a full resequencing data set to infer human population size changes. Proc Natl Acad Sci U S A 102: 18508–18513.

[19] Wiuf C, Hein J (2000) The coalescent with gene conversion. Genetics 155: 451–462.

[20] Yoshino M, Sagai T, Lindahl KF, Toyoda Y, Shiroishi T, et al. (1994) No dosage effect of recombinational hotspots in the mouse major histocompatibility complex. Immunogenetics 39: 381–389.
